# Exploratory, multicenter, open-label study to evaluate the effects of linaclotide in patients with chronic constipation with an insufficient response to magnesium oxide: A study protocol

**DOI:** 10.1016/j.conctc.2022.101019

**Published:** 2022-10-28

**Authors:** Tsutomu Yoshihara, Takaomi Kessoku, Tomohiro Takatsu, Noboru Misawa, Keiichi Ashikari, Akiko Fuyuki, Hidenori Ohkubo, Takuma Higurashi, Michihiro Iwaki, Takeo Kurihashi, Machiko Nakatogawa, Koji Yamamoto, Izuru Terada, Yusuke Tanaka, Atsushi Nakajima

**Affiliations:** a Department of Gastroenterology and Hepatology, Yokohama City University School of Medicine, Yokohama, Kanagawa, 236-0004, Japan; b Department of Palliative Medicine, Yokohama City University Hospital, Yokohama, Kanagawa, 236-0004, Japan; c Department of Internal Medicine, Kanagawa Dental University Yokohama Clinic, Yokohama, Kanagawa, 221-0835, Japan; d Department of Primary Care and General Medicine, Tokyo Medical University, Tokyo, 160-8402, Japan; e Department of Internal Medicine, NamikiKoiso-medical Clinic, Yokohama, Kanagawa, 236-0005, Japan; f Department of Data Science, Yokohama City University School of Medicine, Yokohama, Kanagawa, 236-0004, Japan; g Operational Excellence, Medical Affairs, Astellas Pharma Inc., Tokyo, 103-8411, Japan; h Data Science, Development, Astellas Pharma Inc., Tokyo, 103-8411, Japan

**Keywords:** Chronic constipation, IBS-C, Magnesium oxide, Linaclotide, JPAC-QOL

## Abstract

**Background:**

Chronic constipation leads to poor quality of life, and treatment remains unsatisfactory for patients. In Japan, magnesium oxide has been commonly used as the first choice of treatment for constipation; however, there are some cases of low satisfaction with magnesium oxide treatment. Linaclotide has recently been used to treat chronic constipation. In this study, we will examine whether linaclotide improves symptoms and quality of life in patients showing insufficient response to magnesium oxide.

**Methods:**

This is an exploratory multicenter open-label study. The target number of patients is 64: 32 patients with and 32 without abdominal symptoms. Patients with chronic idiopathic constipation or irritate bowel syndrome with constipation diagnosed according to the Rome-IV criteria are eligible. Patients prescribed 0.99–2 g/day of magnesium oxide for at least 4 weeks will be included. Those who consent to the study will continue taking magnesium oxide for 2–4 weeks, and defecation will be documented. Patients who meet the criteria will be prescribed linaclotide (0.5 mg) daily for 12 weeks. The primary endpoint is a change in the Japanese version of the Patient Assessment of Constipation Quality of Life (JPAC-QOL) score after 12 weeks of treatment.

**Conclusion:**

This is the first study to investigate the usefulness of linaclotide as a second-line treatment for chronic constipation. We will test the efficacy of treatment of constipation in patients with inadequate response to magnesium oxide.

**Trial registration:**

This study is registered with the Japan Registry of Clinical Trials (jRCT, jRCTs031200048).

## 1 Introduction

The prevalence of chronic constipation in adults is estimated at an approximate 15%, and its incidence increases with age [1,2]. Patients with irritable bowel syndrome (IBS) show no organic abnormalities in the small or large intestine, although abnormal bowel movements and uncomfortable abdominal symptoms persist. IBS with constipation (IBS–C) presents with chronic constipation accompanied by abdominal pain and discomfort and is referred to as chronic constipation along with chronic idiopathic constipation (CIC). Patients with chronic constipation have decreased quality of life and low treatment satisfaction [3,4]. Therefore, appropriate medical treatment is required for patients whose quality of life remains impaired by treatment.

In Japan, magnesium oxide (MgO) has long been used to treat chronic constipation. It is classified as an osmotic laxative that facilitates defecation by promoting water secretion in the intestinal lumen and softens the stool. With as many as 10 million prescription users, MgO is widely prescribed in Japan because it is inexpensive, and its dosage can be adjusted by the patients themselves. However, some patients, especially those with renal dysfunction and the elderly, develop hypermagnesemia after taking MgO, which is why it is recommended that their serum magnesium levels be periodically measured [5]. Therefore, prescribing MgO to these patients requires special attention.

Linaclotide is a 14-amino acid peptide guanylate cyclase-C (GC-C) receptor agonist that increases cyclic guanosine monophosphate (cGMP) levels in the intestinal epithelial cells and causes chloride secretion into the lumen, thereby promoting water secretion and bowel movements. It also improves intestinal hypersensitivity, thereby relieving abdominal pain and discomfort [6]. Linaclotide was approved by the Japanese Ministry of Health, Labour, and Welfare in 2016 for use in patients with IBS-C, with an additional indication for chronic constipation in 2018. Linaclotide can be used as a secondary treatment for chronic constipation. Although many patients in Japan take MgO, cases of ineffectiveness have been observed. Clinical trials that evaluated the efficacy of linaclotide as a first-line treatment, such as those reported by Lembo et al. [7], have been conducted previously; however, cases of inadequate response to conventional laxatives have not been reported. Herein, we describe the protocol for our study on the efficacy and safety of switching to linaclotide as a secondary treatment for 12 weeks in patients who show an inadequate response to primary treatment with MgO.

## 2 Methods

### 2.1 Trial design

This is a multicenter, open-label, exploratory study to evaluate the efficacy of linaclotide in patients diagnosed with CIC or IBS-C and inadequately responding to MgO. The study will be conducted at Yokohama City University Hospital, Kanagawa Dental University Yokohama Clinic, and Namiki Koiso Medical Clinic. The study will be managed by the YCU Center for Novel and Exploratory Clinical Trials (Y-NEXT); the administrative office is located at Yokohama City University. Data will be entered and collected by an electronic data capture (EDC) system.

### 2.2 Ethical considerations and trial registration

This study will be conducted in accordance with the principles of the Declaration of Helsinki and approved by the Ethics Committee of Yokohama City University on April 24, 2020 (CRB19-013); it was registered in the Japan Registry of Clinical Trials (jRCT) on June 9, 2020 (jRCTs031200048). All the patients will provide written informed consent. The results obtained from this study will be promptly published through conference presentations or journal article submissions. When publishing the results, necessary measures will be taken to protect the human rights of the research subjects and others involved or the rights and interests of the researchers and others involved.

### 2.3 Drug supply

Linaclotide provided by Astellas Pharma Inc. will be used in this study. The principal investigator at each site and study medication manager will store and manage the study medication in accordance with the study medication management protocol. The subjects will be instructed to take linaclotide (0.5 mg) once daily. Medication adherence will be monitored using a patient diary and any remaining medication that the patients bring with them.

### 2.4 Sample size estimation

The target number of cases in the study is 64. Of these, 32 subjects with abdominal symptoms and 32 without abdominal symptoms will be recruited. This is an exploratory study owing to the lack of data on the Japanese version of the Patient Assessment of Constipation Quality of Life questionnaire (JPAC-QOL) with linaclotide administration. In addition to the primary objective of evaluating the drug response in the overall population, the secondary objective is to evaluate the effectiveness in subgroups based on abdominal symptoms (with and without). The number of patients in each subgroup will be set to ensure 90% statistical power when a paired *t*-test is used at a two-sided significance level of 5% for assessing the change in the JPAC-QOL total score between the baseline and 12 weeks after treatment. A 90% power for each subpopulation will ensure that the main analysis, or the analysis for the total population, has a power of 90% or more if the effect size assumed for each subpopulation is correct. A long-term study of elobixibat in patients with chronic constipation showed that the mean age was 43.9 years and the mean change (standard deviation) in the JPAC-QOL overall score from baseline to week 12 was −0.69 (0.68) [8]. We will consider equivalent or better efficacy of a long-term study of elobixibat as clinically meaningful and set a target number of patients. Miller et al. reported that age is inversely correlated with baseline JPAC-QOL total scores [9]. Considering that the average age of the patients in this study is expected to be approximately 60 years, and assuming a 33% decrease in the mean change in the JPAC-QOL responses and no changes in the standard deviation, the mean change in the JPAC-QOL overall score from baseline to week 12 was assumed as −0.46 (0.68). Under this assumption, 25 subjects would be needed in each subpopulation to detect a significant change from baseline in the JPAC-QOL total score at week 12, with a power of 90% or greater. Assuming a 20% dropout rate during the study period, the target number of patients in each subpopulation in this study is 32. Therefore, the overall target number of cases is calculated as 64.

### 2.5 Eligibility criteria

Patients with CIC and IBS-C will be evaluated according to the Rome-IV criteria [10]. The principal investigator or sub-investigator will check the inclusion and exclusion criteria listed in Table 1 and determine whether the patients meeting the inclusion criteria are eligible. Patients enrolled at this point will be checked at visit 2 to determine whether they meet the medication initiation criteria. Patients who do not meet the medication initiation criteria will be excluded from the study. If they meet the medication initiation criteria, linaclotide prescription will be started.

**Table 1:** Criteria for inclusion, medication initiation, and exclusion.

**Eligibility criteria**	
**Inclusion criteria**	**Exclusion criteria**
1. Written consent is obtained from the patient.	1. Patients with a history of surgical resection of the stomach, gall bladder, small intestine, or colon. However, appendicitis and benign polypectomy are excluded.
2. Patients diagnosed with CIC or IBS-C based on Rome-IV criteria and prescribed MgO 0.99 g–2 g per day for at least 4 weeks.	2. Patients with a history of or currently suffering from inflammatory bowel disease such as Crohn's disease or ulcerative colitis
3. Outpatients whose age at the time of obtaining consent is 20 years or more but less than 85 years.	3. Patients with a history of or currently suffering from ischemic colitis.
4. Patients who are not likely to become pregnant or who agree not to become pregnant until 4 weeks after the last dose of linaclotide.	4. Patients currently suffering from infectious enteritis.
	5. Patients currently suffering from hyperthyroidism or hypothyroidism.
	6. Patients with obvious mechanical obstruction (bowel obstruction due to hernia, etc.).
**Medication initiation criteria**	7. Patients with megacolon or megarectum.
1. Patients with a mean JPAC-QOL total score of 1.0–2.5 at the beginning of the treatment.	8. Patients currently suffering from constipation due to recto-anal dysfunction.
2. Patients who complete all items in the patient diary during the pre-observation period for at least 10 days (at least 5 days per week) during the 14 days prior to the start of treatment.	9. Patients currently suffering from medication-induced constipation.
3. Patients who had taken 0.99 g–2 g of MgO per day for more than 10 days during the 14 days before the start of treatment in the patient diary of the previous observation period.	10. Patients currently suffering from constipation due to organic diseases.
4. Patients who did not excrete “soft (muddy) or watery stools (BSFS type 6 or 7)” during the 14 days prior to the start of treatment and on the day of the start of treatment in the patient diary of the observation period and in the interview on the day of the start of treatment. Patients with diarrhea on the same day (each administration day) and the next day (the day after the end of the administration day) of the use of rescue medication (sennosides, bisacodyl, and sodium picosulfate) by rescue medication criteria will be excluded.	11. Patients currently suffering from active peptic ulcer.
5. Patients who excreted smooth and soft sausage-like stools (BSFS 4) at a frequency of less than twice a week for 14 days prior to the start of treatment in the patient diary of the observation period. This excludes the day on which the rescue medications (sennosides, bisacodyl, and sodium picosulfate) were used by rescue medication criteria (the day of each dose) and the day after (the day after the end of dose).	12. Patients with severe depressive or anxiety symptoms that are judged to affect the assessment of drug efficacy.
6. Patients who have not used or performed any rescue medication, concomitantly prohibited drug, therapy, or prohibited test since enrollment. Subjects who meet the criteria for rescue medication and have used the rescue medication (sennosides, bisacodyl, sodium picosulfate) less than or equal to two consecutive times will not be excluded.	13. Patients with a history of drug or alcohol abuse or current abuse within one year prior to obtaining consent.
7. Patients who did not use the rescue medication from the day before the start of treatment to the start of treatment.	14. Patients who have used or are planning to use the following prohibited drugs: Lubiprostone, elobixibat, polyethylene glycol (Macrogol 4000), and lactulose
8. Patients for whom the investigator or sub-investigator has not judged that it is inappropriate to start administration.	15. Patients who have been prescribed or will be prescribed antiparkinsonian, antipsychotic, antimanic, or psychostimulant medication at the time of enrollment.
	16. Patients who have undergone or are scheduled to undergo gastrointestinal endoscopy within 3 days prior to enrollment.
	17. Patients who have had or currently have a malignant tumor of the digestive system (excluding gastrointestinal cancers that have been cured by endoscopic treatment).
	18. Patients currently suffering from serious cardiovascular, respiratory, renal, hepatic, gastrointestinal, hematological, neurological or psychiatric diseases.
	19. Patients with a history of drug allergy.
	20. Patients who have participated or are currently participating in clinical trials, post-marketing clinical trials, or clinical research for other ethical drugs or medical devices within 12 weeks prior to obtaining consent.
	21. Patients who are judged inappropriate to participate in this study.

### 2.6 Study flow

**Fig. 1:**
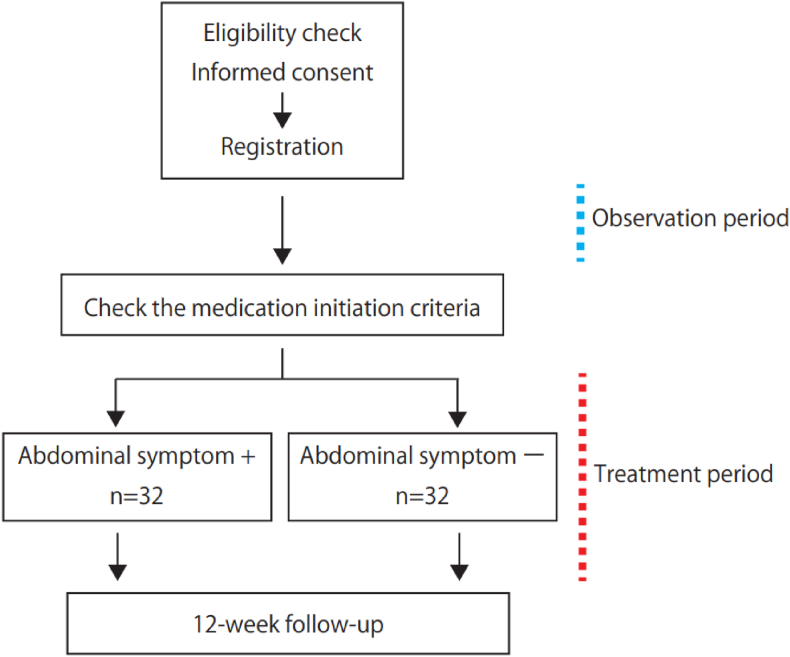
Study flow.

The flow of the study is shown in Fig. 1. The principal investigator or sub-investigator will check the eligibility criteria at visit 1 and medication initiation criteria at visit 2 (Table 1). During the observation period, patients will ingest a prescribed dose of 0.99 g–2 g of MgO. Patients who meet the medication initiation criteria will be asked to complete the blood tests and questionnaires provided in Table 2. During the medication period, MgO will be discontinued and linaclotide will be administered at a dose of 0.5 mg per day, which will be reduced or withdrawn depending on the symptoms of defecation. If there is no defecation while taking the study medication, the use of rescue medication will be acceptable according to the following protocol: the study medication is prescribed for each visit, and any remaining medication is collected by the physician in charge. The duration of medication will be 12 weeks.

**Table 2:**
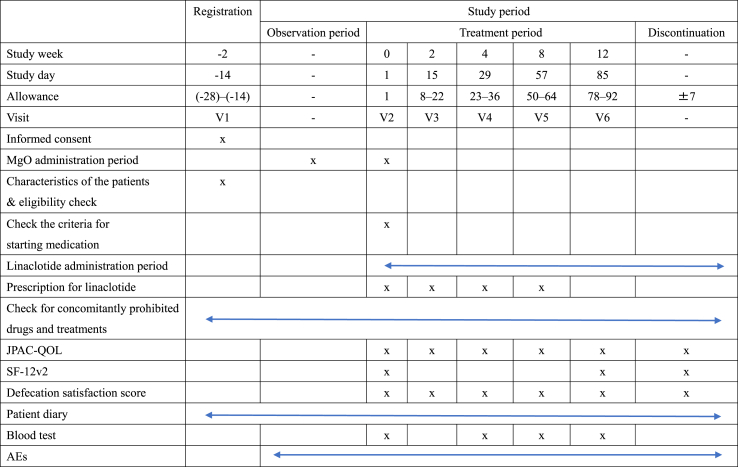
Schedule of the study..

#### 2.6.1 Use of rescue medication

If there is “no defecation” for more than 3 days during the study period, the subject can take one type of medication according to the dosage and administration as directed by the principal investigator or sub-investigator. If there is no or insufficient defecation the following day, the subject can take another dose of rescue medication. If the second dose of rescue medication does not produce a bowel movement or is insufficient, the subject can use a rescue medication or enema according to the dosage and administration. The use of rescue medications or enemas will be documented in the patient's diary. The rescue medications include sennosides, bisacodyl, and sodium picosulfate. However, if the drug is used without meeting the criteria for rescue medication, it will be considered a prohibited medication.

#### 2.6.2 Concomitant prohibited treatments

During the study period, the use of the drugs, treatments, and tests listed in Table 3 is prohibited.

**Table 3:** Concomitant medications, treatments, or tests that are prohibited during the study.

**Drugs prohibited in combination with**

【**Ethical pharmaceuticals**】
1. Sennosides, bisacodyl, sodium picosulfate (when used within 3 days of non-defecation during the study period, and excluded when used under the rescue medication criteria)
2. Lubiprostone, elobixibat, polyethylene glycol (Macrogol 4000), lactulose
3. Anticholinergic drugs
4. Rhubarb
5. Antidiarrheals
6. Laxatives other than 1. and 2. (except for MgO in the observation period and on the first day of treatment period)
7. Narcotic analgesics, narcotics, and narcotic antitussives (narcotic antitussives are allowed on an ad hoc basis)
8. Antiparkinsonian drug
9. Antipsychotic drug
10. Antimanic drug
11. Psychostimulant
**【Over-the-counter drug】**
OTC drugs indicated for constipation, diarrhea, or abdominal pain

**Tests/treatments prohibited during the study period**
1. Enemas (use of enemas during the study period without bowel movements after two doses of rescue medication falls under the category of prohibited concomitant medications)
2. Defecation
3. Gastrointestinal endoscopy

#### 2.6.3 Dose reduction and withdrawal criteria

If a subject experiences diarrhea (BSFS 6 or 7), the dose of linaclotide will be reduced to 0.25 mg once daily; if diarrhea persists after the dose reduction, the drug will be withdrawn as directed by the principal investigator or sub-investigator.

#### 2.6.4 Dose escalation and resumption criteria

During the treatment period, the dose can be increased again to 0.5 mg in cases where it had been reduced to 0.25 mg once daily. If the subject experiences at least one BSFS type 1 to 5 during the withdrawal period, linaclotide will be resumed the following day, as directed by the principal investigator or sub-investigator.

### 2.7 Discontinuation of research on individual subjects

The study will be discontinued in study subjects when any of the following criteria are met:

(1) when it is judged difficult to continue the study due to the occurrence of adverse events and ischemic colitis, or inflammatory bowel disease is suspected after administration; (2) when the subject requests discontinuation of participation in the study; (3) when it is impossible to continue the study due to the subject's circumstances, such as no longer coming to the hospital, moving, or being too busy; (4) when the subject does not meet the criteria for initiation of treatment within 28 days of enrollment, or when it is found after the start of treatment that the subject does not meet the eligibility criteria or he/she meets the exclusion criteria, and it is determined that continuation of the study is inappropriate; (5) when the subject's disease worsens or the improvement with the study drug is insufficient and the principal investigator or sub-investigator determines that the subject is not suited to the treatment; and (6) pregnancy after the start of treatment.

### 2.8 Observation and handling of adverse events

In the event of an illness, the principal investigator or sub-investigator will take appropriate measures to ensure the safety of the study subjects, including discontinuation of treatment and administration of the study drug. If treatment is necessary, the investigator will inform the study participants of its need. If an adverse event is continued even at the last observation at the end of the study period, the investigator or sub-investigator continues to follow up until clinical stability or return to the baseline state is achieved.

### 2.9 Evaluation items

Efficacy after switching from MgO to linaclotide will be evaluated using the JPAC-QOL scale.

The defecation satisfaction score is a 4-point scale (satisfactory, somewhat satisfactory, somewhat unsatisfactory, and unsatisfactory) that indicates the subject's level of satisfaction upon defecation.

The severity score of each abdominal symptom (abdominal distention, pain, and discomfort) will be evaluated on a 5-point scale (1: none, 2: weak, 3: moderate, 4: strong, and 5: very strong).

The severity of constipation is rated on a 5-point scale (0: none (no symptoms of constipation), 1: mild (slight symptoms of constipation), 2: moderate (constipation, but not severe), 3: severe (severe constipation and difficulty in defecating or only a slight feeling of defecation after going to the toilet), and 4: extremely severe (stubborn constipation, almost no defecation, or little or no sensation of defecation when going to the toilet)).

Stool shape (BSFS) will be evaluated on a 7-point scale (1: hard and colicky rabbit droppings (difficult to defecate); 2: sausage-like but bumpy (lumpy); 3, sausage-like with cracks on the surface; 4, smooth and soft sausage-like or snake-like with a coiled surface; 5, soft and semi-soft stools with distinct ends; 6, irregularly shaped small pieces of stool with loose edges and squishy, muddy stools, and 7: watery, liquid stools with no solids).

Straining in stool will be evaluated on a 5-point scale (1: not at all, 2: a little, 3: moderately, 4: a lot, and 5: extremely).

### 2.10 Endpoints

#### 2.10.1 Efficacy endpoints

The primary endpoint is the change from baseline in terms of the JPAC-QOL total score after 12 weeks of treatment. The secondary endpoints are listed in Table 4.

**Table 4:** Study endpoints.

Study endpoints	
**Primary endpoint**	**Secondary endpoints**
Change in JPAC-QOL score from baseline at 12 weeks of linaclotide treatment	1. Change in weekly average of SBM frequency
	2. Percentage of subjects with SBM within 24 h of first dose
	3. Time to first SBM
	4. Change in weekly average of CSBM frequency
	5. Percentage of subjects with CSBM within 24 h of first dose
	6. Change in weekly average of stool forms
	7. Average weekly change in abdominal bloating severity score
	8. Average weekly change in abdominal pain and abdominal discomfort severity score
	9. Average weekly change in severity score of straining
	10. Change in JPAC-QOL overall score and subscale scores
	11. SF-12v2 summary score, change in subscale
	12. Satisfaction score on defecation

#### 2.10.2 Safety assessment

Safety endpoints include adverse events that occur in the study subjects as a result of their participation in the study. Adverse events are all unwanted or unintended injuries or illnesses or signs thereof (including abnormal laboratory values) that occur in the study subjects, regardless of any causal relation. This includes any exacerbation of a pre-existing condition (not including the underlying disease) during the study period. The investigator or principal investigator will evaluate the extent, causal relationship, and severity of the adverse events following the Common Terminology Criteria for Adverse Events v5.0.

A “serious illness” is defined as any of the following: (1) death; (2) diseases that may lead to death; (3) diseases that require hospitalization or prolonged hospitalization for treatment; (4) disability; (5) diseases that may lead to disability; (6) diseases that are as serious as (3) through (5) above and diseases that may lead to death; and (7) congenital diseases or anomalies in later generations.

### 2.11 Eligibility for analysis

The population to be analyzed is defined as follows: the primary analysis population is the full analysis set (FAS). FAS is defined as all patients enrolled in the study receiving at least one dose of linaclotide and showing at least one efficacy endpoint measured after baseline.

Additionally, the target population (per-protocol set: PPS) that meet the study protocol is defined as those subjects in the FAS who complied with the following criteria: (1) primary endpoints are available. (2) Inclusion criteria are met. (3) Exclusion criteria are not met. (4) The medication initiation criteria are met. (5) The study drug is administered for at least 42 days from the start of treatment or the day following the start of treatment. However, if the primary reason for discontinuation is “inadequate response” or “worsening of the target disease,” even if less than 42 days elapsed, the subject will be considered for PPS. (6) The drug compliance rate, excluding drug withdrawal during the treatment period, is at least 5/7 × 100%. (7) No rescue treatment or prohibited concomitant treatment is administered during the treatment period. (8) No prohibited tests are performed during the treatment period.

The safety analysis set (SAS) is defined as the population enrolled in the study who received the study drug at least once.

### 2.12 Statistical analysis

Efficacy analyses will be performed using FAS. The results of the statistical tests will be interpreted according to the FAS; PPS will be used to assess the robustness of the results based on the FAS. The primary endpoint is the change in the JPAC-QOL total score from baseline at week 12. A paired *t*-test will be performed at a two-sided significance level of 5% for the change in the JPAC-QOL total score between the baseline and week 12. Additionally, the point estimation and 95% confidence intervals for the mean change will be calculated.

As secondary analyses, subgroup analyses will be performed for the following factors:

(1) Abdominal symptoms (with or without abdominal symptoms)
(2) Gender (male, female)
(3) Age (<65 years, ≥65 years)

The secondary endpoints are presented in Table 4. Summary statistics of the measured values and changes from baseline will be calculated for each time point for items 1, 4, and 6–11. Paired t-tests will be performed on the amount of change, and 95% confidence intervals for differences will be calculated. For items 2 and 5, the number and percentages of subjects as well as the 95% confidence interval will be calculated. For item 3, a Kaplan-Meier curve will be plotted, and the median and 95% confidence interval of the time to event will be calculated. The earliest discontinuation, failure to observe, and expiration during the observation period will be defined as censoring. For item 12, the number and percentages of patients in each category will be calculated at each time point.

For safety analysis, the number of cases and percentages of adverse events and adverse events that cannot be denied as related to the study drug will be calculated. For the measurable parameters in the clinical laboratory tests, summary statistics of the actual measured values and changes from baseline will be calculated for each time point using descriptive statistics. For countable parameters, a frequency tally will be performed at each assessment time point. A shift table of the laboratory measurement abnormalities observed at each time point from the baseline compared to the normal range during the treatment period will be recorded.

### 2.13 Data management and monitoring

A study subject identification code will be assigned when the patients are enrolled. The study subject identification code consists of a numerical code or other symbols unrelated to information that can help identify specific individuals, such as their initials or medical record IDs. The information will be anonymized using the study subject identification code when preparing documents related to this study, such as case registration forms and report forms (CRF). The principal investigator will prepare a correspondence list containing information such as the name and medical record ID of the study subjects so that they can be identified from the anonymized information as necessary. This information will be strictly stored and managed so that it is not leaked. Additionally, the principal investigator strictly will store and manage consent forms, medical records, examination data, patient diaries, and medical questionnaires in a lockable storage room at each facility. Electronically stored data in an electromagnetic storage device, such as a PC or USB memory stick that is independent of the hospital LAN or the Internet, will be password-protected, stored, and managed strictly in a lockable storage facility when not in use.

The CRF used in this study will be stored in the EDC system (VIEDOC™, Viedoc Technologies AB, Uppsala, Sweden). The principal investigator will submit the CRF by entering the data into the EDC system immediately after the completion of the examination and observation of each subject.

Monitoring will be outsourced to an external agency and planned accordingly.

## 3 Discussion

This is the first study to evaluate the efficacy and safety of switching to linaclotide in patients with CIC or IBS-C who are taking MgO and show an inadequate response. In Japan, the Ministry of Health, Labour, and Welfare recommends the use of linaclotide after the use of conventional laxatives with inadequate efficacy (https://kouseikyoku.mhlw.go.jp/shikoku/iryo_shido/000072356.pdf). However, no studies on the efficacy or safety of linaclotide as a second-line treatment for CIC or IBS-C have been conducted yet. Additionally, the clinical trial of linaclotide that preceded the present study included a large number of young patients. For example, Fukudo et al. report the efficacy of linaclotide in patients with chronic constipation, wherein the mean age of the subjects was 42.7 years (±11.7 SD) and 3.4% of all subjects were over 65 years [11]. In Japan, linaclotide is started at 0.5 mg. In a randomized controlled trial where low-dose linaclotide was administered orally for chronic constipation, the mean age of the subjects was less [7,12]. The morbidity associated with chronic constipation increases with age [2]. Patients with IBS have a lower average age than patients with CIC, and the prevalence of IBS is approximately 10% with IBS-C accounting for approximately 20–30% of these patients [13]. Considering that the reported prevalence of CIC is 14% [14], it is likely that the prevalence of IBS-C will be lower than that of CIC and the overall age of the patients will be relatively high in this study, as many elderly people with a diagnosis of CIC will be included in the study. Therefore, these trials have discrepancies in clinical practice. Since this study is expected to attract more elderly patients and patients with underlying diseases as compared to previous clinical trials, we believe that the data gap between clinical trials and clinical practice will be eliminated, thus providing efficacy and safety data in patients that are similar to those in actual clinical practice.

In an internet survey of patients with chronic constipation in the U.S. and Japan, many respondents reported abdominal symptoms, with abdominal discomfort and bloating being the most bothersome symptoms [15,16]. Epidemiological studies conducted in the U.S. and Japan have also reported that patients with chronic constipation have reduced health-related quality of life (SF-12v2) and labor productivity as compared to healthy individuals [3,17]. Studies involving patients with IBS-C and CIC have also shown that of the large number of patients treated with over-the-counter and prescription drugs, many are not satisfied with their effectiveness and suffer from inadequate efficacy and side effects such as diarrhea [18,19]. The results of these previous studies suggest that constipation symptoms perceived as bothersome, such as abdominal bloating, may reduce the quality of life of patients with CIC and IBS-C. Furthermore, as linaclotide administration may lead to changes in patients’ symptoms and alter their quality of life, quality of life is considered the primary endpoint of this study. The Patient Assessment of Constipation Quality of Life Questionnaire (PAC-QOL), developed to assess the impact of constipation symptoms on quality of life, is widely used internationally as a standard disease-specific measure for chronic constipation. In addition, PAC-QOL has been used in studies that have included patients with IBS-C [20]. In Japan, the reliability and validity of the Japanese version of the PAC-QOL (JPAC-QOL) have been evaluated [21,22]. However, the only drug developed using JPAC-QOL in Japanese clinical trials for the treatment of chronic constipation is elobixibat; linaclotide has no JPAC-QOL data for the quality of life [8,23]. Therefore, JPAC-QOL is considered the primary endpoint in this study.

The strengths of this study are that it is the first to use linaclotide as a second-line treatment for MgO and is expected to provide data closer to clinical practice as a large proportion of elderly patient enrollment is anticipated. The limitations of this study are that it is an exploratory trial instead of a randomized trial, and the sample size is small.

## Funding

This study is funded by 10.13039/501100004948Astellas Pharma, Inc. (Tokyo, Japan). Astellas is not involved in the collection or analysis of the study results.

## Author's contributions

TY, TH, IT, YT, and AN designed this study. TY, TKe, TT, NM, KA, AF, HO, TH, MI, TKu, and MN will recruit patients and follow them up. The results will be analyzed and interpreted by TY, Tke, KY, and AN. All the authors have read and approved the manuscript.

## Data sharing statement

Data are available upon reasonable request.

## Declaration of competing interest

The authors declare the following financial interests/personal relationships which may be considered as potential competing interests: This study is funded by Astellas Pharma Inc., the distributor of linaclotide. Astellas Pharma Inc. is involved in the drafting of the research protocol, obtaining consent forms, and managing the drugs and their delivery to the institutions.
